# Zika Virus Targeting by Screening Inhibitors against NS2B/NS3 Protease

**DOI:** 10.1155/2019/3947245

**Published:** 2019-11-22

**Authors:** Hani Choudhry, Faisal A. Alzahrani, Mohammed A. Hassan, Asma Alghamdi, Wesam H. Abdulaal, Muhammed A. Bakhrebah, Mazin A. Zamzami, Nawal Helmi, Fawzi F. Bokhari, Mustafa Zeyadi, Othman A. Baothman, Mohammad A. Kamal, Mohiuddin K. Warsi, Ashraf Ali, Bushra Jarullah, Mohammad S. Jamal

**Affiliations:** ^1^Department of Biochemistry, Cancer Metabolism and Epigenetic Unit, Faculty of Science, Cancer and Mutagenesis Unit, King Fahd Medical Research Center, King Abdulaziz University, Jeddah 21589, Saudi Arabia; ^2^Department of Biological Sciences, Rabigh College of Science and Arts, King Abdulaziz University (Jeddah), Rabigh Campus, Jeddah, Saudi Arabia; ^3^Life Science and Environment Research Institute, National Center for Genome Technology, KACST, Riyadh 12371, Saudi Arabia; ^4^Department of Applied Biochemistry, Faculty of Sciences, University of Jeddah, Jeddah, Saudi Arabia; ^5^Center for Health Studies, Armed Forces Hospitals, Taif Region, Taif, Saudi Arabia; ^6^Department of Biochemistry, Faculty of Sciences, University of Jeddah, Jeddah, Saudi Arabia; ^7^University of Jeddah Centre for Science and Medical Research (UJ-CSMR), Jeddah, Saudi Arabia; ^8^King Fahd Medical Research Center, King Abdulaziz University, Jeddah 21589, Saudi Arabia; ^9^Department of Science of Agriculture, Food and Environment (SAFE), University of Foggia, Via Napoli 25, 71122 Foggia, Italy; ^10^Department of Biotechnology, Kadi Sarva Vishwavidyalaya, Gandhinagar, Gujrat, India

## Abstract

Zika flavivirus is suspected to cause Guillain-Barre syndrome in adults and microcephaly, along with other congenital abnormalities in infants. Presently, no vaccines or therapeutics are available. Here, we report novel compounds identified by high-throughput virtual screening of Maybridge chemical database and molecular docking studies. We selected viral enzyme NS2B/NS3 serine protease as the therapeutic target because of its important role in viral replication. We selected seven potential compounds as antiviral drug candidates because of their high GOLD fitness score, high AutoDock Vina score, or X-Score binding energy and analyzed the strength of molecular interactions between the active site amino acids and selected compounds. Our study also provides a foundation for similar studies for the search of novel therapeutics against Zika virus.

## 1. Introduction

Zika virus (ZIKV) is a reemerging mosquito-borne pathogen that belongs to the family Flaviviridae and genus *Flavivirus* [1]. The other members of this family include dengue virus, West Nile virus, Japanese encephalitis virus, and yellow fever virus [2]. ZIKV was first accidentally isolated in 1947 by the Uganda Viral Research team from a sentinel rhesus monkey in the Zika Forest while searching for yellow fever virus [3]. Later in 1948, the same virus was isolated from *Aedes africanus* mosquitoes [4]. Humans serve as the amplifying host for ZIKV, while *Aedes* mosquitoes are the vectors transmitting the pathogen. The first Zika virus infection was reported in 1954 in a young girl in Nigeria [5, 6]. Later, the virus travelled to Asia and was first identified in *Aedes aegypti* mosquitoes in Malaysia in 1966 [7], while the first human infection was reported in Java, Indonesia, in 1977 [8]. Until 2007, ZIKV was not considered a significant human pathogen. However, an outbreak of fever and rash caused by this virus in the Yap Islands changed this notion. This small outbreak had 49 confirmed cases and more than 50 probable cases of ZIKV infection in humans [9]. A couple of years later, a second larger outbreak took place in French Polynesia, where 333 cases were confirmed, and more than 19000–32000 suspected cases were observed. It was during this period that the relationship between ZIKV infection and Guillain-Barre syndrome was first noticed [10]. During subsequent outbreaks in Brazil in 2014-15, more evidence was collected pointing towards the possible connection of congenital brain anomalies and microcephaly in children exposed to ZIKV infection [11, 12].

The mode of transmission of infection is either through the mosquito, *Aedes*, or blood transfusion from Zika-infected patients. In some cases, it was found that the Zika infection spread through sexual contact. It was found in the infected patients that the virus persisted strongly in semen longer than in blood, urine, and saliva [13–15]. Infected women may also transmit the virus to her growing fetus or to the baby during birth. Therefore, there is strong evidence that the ZIKA infection can spread from human to human by different methods, such as blood transfusion, sexual contact, and from mother to fetus.

WHO announced ZIKV as a public health emergency of international concern in February 2016. Since then, researchers worldwide have tried to understand this virus and its pathogenesis more deeply. To this date, there are no vaccines or antiviral drugs to prevent or treat ZIKV infections, though many vaccine candidates are presently under development [16]. Therefore, alternate therapeutics are the need of the hour to prevent and inhibit the spreading of infection, as well as for treatment [17].

Zika virus is an enveloped spherical virus with 10.7 kb single-strand positive-sense RNA genome. Genomic RNA of ZIKV consists of a single open reading frame (ORF), with untranslated regions (UTRs) at 3′ and 5′ ends. A single large polypeptide consisting of 3423 amino acids is encoded by viral ORF which is then cleaved by both viral and host proteases into three structural proteins, namely, envelope (E), premembrane (prM), capsid (C), and seven nonstructural proteins called as NS1, NS2A, NS2B, NS3, NS4A, NS4B, and NS5 [18]. The structural proteins help in the creation of virus particles [19]. The C-reactive protein forms the nucleocapsid after binding to the RNA genome. The prM protein keeps a check at the untimely fusion of viral particles with the host-cell membranes [20]. The E protein is the key player in host recognition via receptor binding and fusion. The host's antibodies also mainly target the fusion loop region of ZIKV E protein [21]. There is a small hidden region called M protein within the E protein. Both these proteins (E and M) form the icosahedral-shaped arrangement in 60 repeating units [19]. The nonstructural proteins of the virus take care of replication, evading host immune responses, assembly, etc. [22]. There are two important nonstructural proteins playing a major role in viral replication: one is NS3, comprising serine protease at its N-terminal, which needs another viral protein NS2 for its activity. The NS3 protein contains RNA helicase and RNA triphosphatase activities at its C-terminal region, which engages in RNA synthesis as well as capping functions [23]. NS2B-NS3 protease has been known for cleaving 31 human proteins, which were identified by unbiased N-terminomics, particularly during Zika virus infection depletion of protein such as autophagy-related protein 16-1 (ATG16L1) and eukaryotic translation initiation factor 4 gamma 1 (eIF4G1), which mediates type-II interferon production and host-cell translation, respectively, possibly involve in immune system evasion and driving the Zika life cycle as well as in mediating neurotoxicity through cleavage of host proteins [24, 25]. ZIKV-NS2A destabilizes the adherens junction complex and results in infection in the developing mammalian brain [26].

The two-protein component system of NS2B/NS3 presents an excellent target for the development of antiviral drugs against ZIKV. The enzyme complex NS2B/NS3 plays an important role in viral replication inside the host, as well as helps the virus in evading innate immunity [27]. NS3 has both a protease domain and an RNA helicase domain at its C-terminal. NS3 protein requires another membrane-bound protein NS2B for proper orientation and function of enzyme [28]. Hydroxychloroquine inhibits NS2B-NS3 protease activity, which in turn results in significantly decreasing Zika virus infection in placental cells [29]. The recent research studies on the development of therapeutics against ZIKV have targeted nonstructural proteins that are essential for the replication of viruses. These include development of inhibitors for NS2B/NS3 protease, in particular. A few researchers have reported different inhibitors against NS2B/NS3 serine protease, though these findings are still in early lead phases [27, 30, 31].

Here, we report seven potential inhibitors against NS2B/NS3 protease identified through high-throughput virtual screening of Maybridge database. Molecular docking studies are also conducted for the protein and selected compound complex. The nature of molecular interactions between the protein (active site amino acids) and the selected compounds is also explored. Our study provides a foundation for the development of new structure-based inhibitors as future drug candidates against Zika virus.

## 2. Materials and Methods

### 2.1. Library Selection

We have selected the Maybridge database for virtual screening. The Maybridge database (over 53,000 compounds) is a commercial library of small hit-like and lead-like compounds. Out of these compounds, the HitCreatorTM Collection (14,400 compounds) represents the diversity covering the drug-like chemical space. Maybridge also has a fragment library (30,000 fragments), a hit-to-lead building block collection, and a rule of 3 at least 2500 diversity fragment library with a Tanimoto similarity coefficient of 0.6.6.

### 2.2. Target Preparation and Virtual Screening

The crystal structure of ZIKA NS2B-NS3 (PDB-ID: 5GXJ) was retrieved from the Protein Data Bank [32]. Protein is prepared using the removal of water molecules followed by addition of hydrogen atoms. The amino acids His1051, Gly1133, Thr1134, Ser1135, and Tyr1150 were used as the active sites for docking experiments. Virtual screening has been performed by using the AutoDock Vina (http://vina.scripps.edu/download.html) and GOLD programs. GOLD (Genetic Optimization for Ligand Docking) is a commercially available docking program produced from the collaboration between the University of Sheffield, GlaxoSmithKline plc, and Cambridge Crystallographic Data Center [33]. It is an automated ligand-docking program that uses a stochastic method with a genetic algorithm to explore the full range of ligand conformational flexibility with partial flexibility of the protein, and it satisfies the fundamental requirement that the ligand must displace loosely bound water on binding. Best scored compounds from the GOLD and AutoDock software were selected for their mode of interaction analyses. Best scored and conformation of compounds from the GOLD and AutoDock software were selected for their mode of interaction analyses. Selecting the promising conformation from the docking study is crucial for determining accurate accuracy of the ligand within the active site of the target [34, 35]. The default setting available in the GOLD and AutoDock software was used to select the best conformation of the ligand.

### 2.3. Postdocking Analysis

X-Score, a consensus scoring function, uses the negative logarithm of the dissociation constant of the ligand to the protein, ÀlogKd, as the average of three scoring functions (HP Score, HM Score, and HS Score) [36]. X-Score was known to have an accuracy of ±2.2 kcal/mol relative to the actual binding energies. For analyzing the interactions of docked protein-ligand complexes, the LIGPLOT programme was used to check the hydrogen bond and hydrophobic interactions between the receptor and the ligand [37]. Also, PyMOL (V-1.3) [38] and Discovery Studio Visualizer (http://accelrys.com/products/collaborative-science/biovia-discovery-studio/visualization-download.php) were used to visualize the interactions and prepare figures for top-ranked molecules.

## 3. Results and Discussion

The *in silico* approach of high-throughput virtual screening has become one of the most popular techniques for drug discovery, currently. With this approach, not only very large amounts of data can be analyzed quickly, but also an immense amount of energy, time, and costs related to drug discovery research can be saved [39].

ZIKV has long been neglected since its first identification, assuming its low/mild pathogenicity in humans. But the recent suggestions of strong links between ZIKV infection with Guillain-Barre syndrome in adults and congenital defects and microcephaly in newborns have brought the attention of scientific fraternity to this virus. Presently, there are no effective treatments available to cure ZIKV infection, and hence, development of novel antiviral therapeutics is urgently needed.

In the present study, we used *in silico* virtual screening and docking approach to identify potential chemical molecules active against the viral protein NS2B/NS3 serine protease complex. These identified molecules may pave way for the development of future antiviral drugs against Zika virus. The Maybridge chemical database was used for virtual screening of lead-like compounds. Docking simulation was performed by using the AutoDock Vina and GOLD docking software. GOLD allows full flexibility of the ligand and partial flexibility of the receptor [40]. The docked compounds were assessed on the basis of the GOLD fitness score. To reduce the chances of false results, only compounds with higher fitness scores were chosen for further studies. To understand likely interactions between lead compounds and proteins, we analyzed the docking results through the GOLD fitness score, constructive binding, and strong interactions of important amino acids of the protein and the compounds. The best compounds selected on the basis of the GOLD fitness score were further analyzed with X-Score. It predicts the binding energies by calculating the negative logarithm of the dissociation constant of the compounds to the protein. It predicts the binding energies with the accuracy of ±2.2 kcal/mol [41]. Boronate inhibitor (PubChem ID: 16740933) was used as a reference compound. Recently, this compound has been used as reference molecules for screening the database against the NS2B-NS3 target [42].

**Figure 1:**
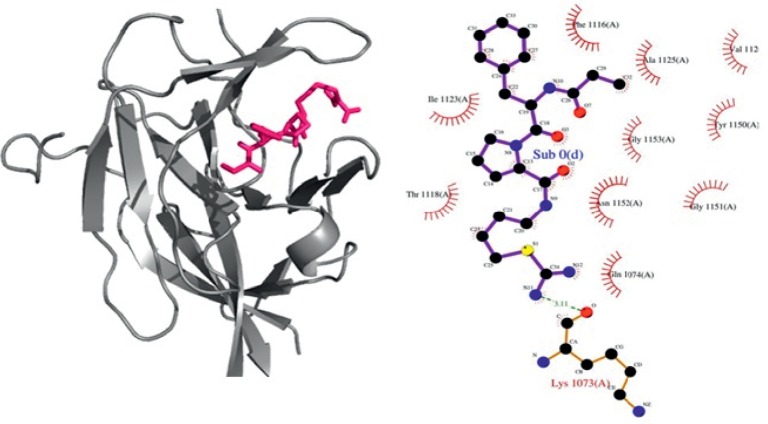
Binding mode of boronate inhibitor (positive control as reference) at the active site of NS3.

**Figure 2:**
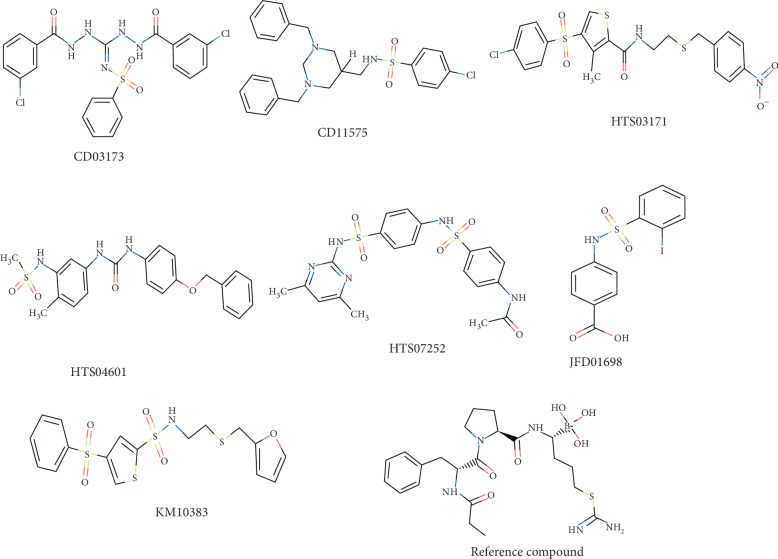
Figure represents 2D structures of the seven best-selected compounds.

On the basis of the docking program's binding affinity, 7 compounds were selected from the Maybridge database having high GOLD fitness scores and binding energies. These compounds have been found to have better binding energy (–6.9 kcal/mol) and have shown strong interactions with active site amino acids (Figure 1). Compounds (except HTS03171 and CD03173) were also found to have drug-like properties [43]. HTS03171 and CD03173 were found to have one Lipinski rule of violation. These compounds have been listed in Table 1.  Figure 2 represented the 2D structures of best-selected compounds. Drug-like properties of the compounds are listed in Table 1.

The highest GOLD fitness scores of 72.01 and 71.21 were shown by CD03173 and JFD01698, respectively (Table 2). CD03173 was found to have binding energy from AutoDock Vina. Compounds HTS07252 and HTS04601 also displayed fitness scores of 69.68 and 69.59, respectively. Compounds CD11575, HTS03171, and KM10383 also formed stable complexes with proteins with fitness scores of 68.67, 67.81, and 62.45, respectively. Moreover, binding energies from AutoDock Vina for these compounds were –8.1, –6.9, and –6.8 kcal/mol, respectively.

**Figure 3:**
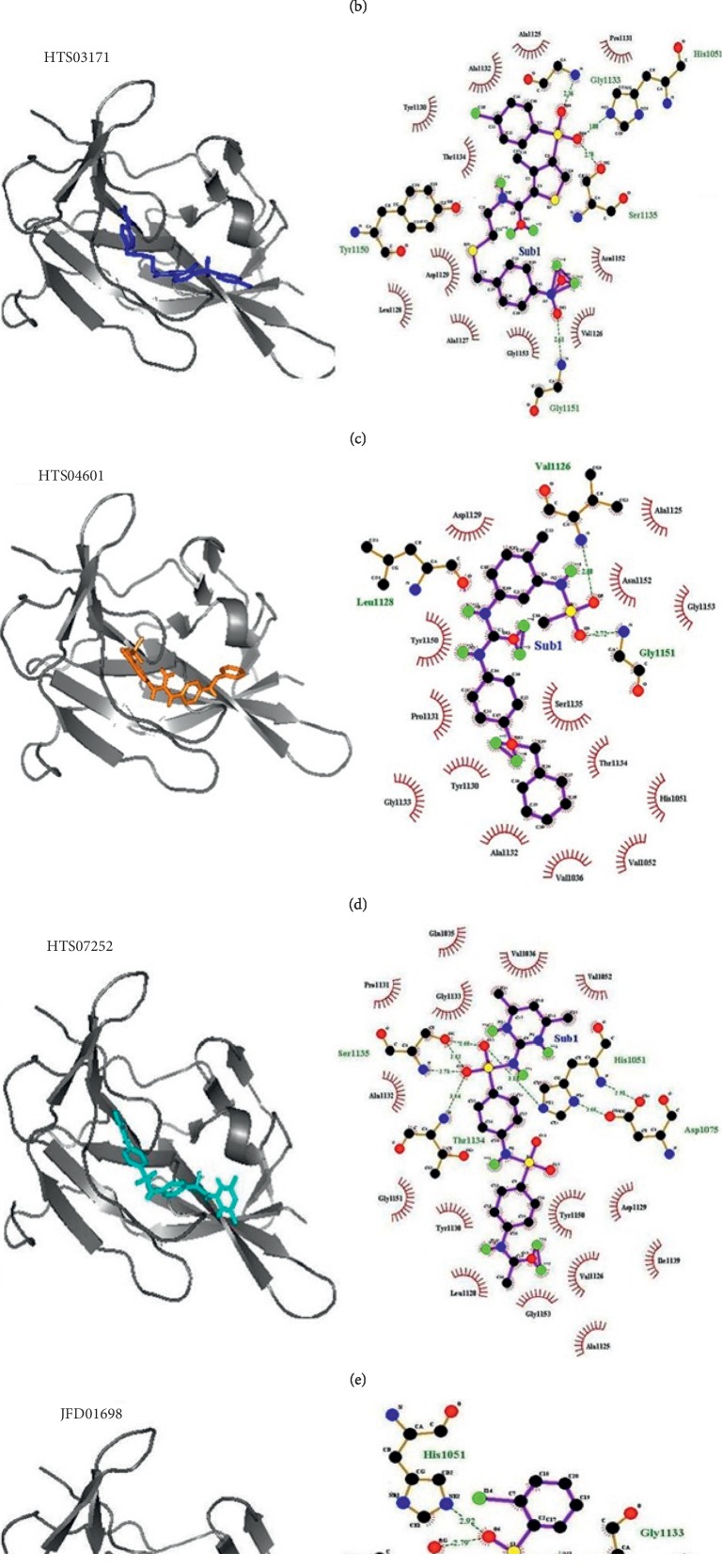
Figure represents the conformation of ligands (selected compounds) at the active site of target NS2B/NS3 serine protease. (a) CD03173. (b) CD11575. (c) HTS03171. (d) HTS04601. (e) HTS07252. (f) JFD01698. (g) KM10383.

Within the protein-ligand complex, hydrogen bonds play an important role in determining its specificity and affinity [44]. To evaluate the affinity of the selected compounds with the enzyme, we also investigated the amino acids of proteins involved in forming hydrogen bonds with the compounds and the strength of these bonds. Amino acids involved in hydrophobic interactions between proteins and ligands were also studied. Hydrogen bonds and hydrophobic interactions play a crucial part in giving shape and stabilizing the protein-ligand complexes [45]. These molecular interactions may be supportive in enhancing proximity of ligand in the active site of protein, thus helping in its biological activity [46]. Thus, we also did a comprehensive analysis of the molecular interactions, hydrogen bonds, and hydrophobic interactions, in particular, within the active site of NS2B/NS3 protein, for the selected compounds (Table 2 and Figure 3).

Compound HTS07252 had a GOLD score of 69.68 and X-Score binding energy –7.4 kcal/mol (Table 1). Four amino acids (His1051, Thr1134, Ser1135, Gly1151) were found to be involved in forming hydrogen bonds with this compound within the range of 2.6.0–3.1.4 Å (Table 2 and Figure 3). In addition to this, eighteen other residues (Gln1035, Val1036, His1051, Ala1125, Val1126, Leu1128, Asp1129, Tyr1130, Pro1131, Ala1132, Gly1133, Thr1134, Ser1135, Ile1139, Tyr1150, Gly1151, Asn1152, Gly1153) are involved in forming hydrophobic interactions within the range of 2.2.7–3.8.7 Å (Table 2 and Figure 3). This compound formed the highest number of non-bonded interactions with the protein.

Compound HTS03171 had a GOLD score of 67.81 and X-Score binding energy –6.5 kcal/mol (Table 1). AutoDock binding energy of this compound was –6.9 kcal/mol. Four amino acids at the active site of NS2B/NS3 enzyme (His1051, Gly1133, Ser1135, and Gly1151) formed hydrogen bonds with it within the range of 2.36–3.08 Å (Table 2 and Figure 3). Fifteen amino acids (His1051, Ala1125, Val1126, Ala1127, Leu1128, Tyr1130, Pro1131, Ala1132, Gly1133, Thr1134, Ser1135, Tyr1150, Gly1151, Asn1152, and Gly1153) participated in the formation of hydrophobic interactions within the range of 2.34–3.89 Å (Table 2 and Figure 3). HTS03171 was found to violate one Lipinski rule (Table 3). However, HTS03171, having less binding energy as compared to boronate inhibitor, the binding mode of interaction was found to be reasonably good (Figure 1).

Compound HTS04601 make complex using AutoDock with binding energy –7.2 kcal/mol. GOLD fitness score and X-Score binding energy were 69.59 and –6.4 kcal/mol, respectively (Table 1). Only two amino acids (Val1126 and Gly1151) formed hydrogen bonds within 2.72–2.88 Å range, and thirteen amino acids (His1051, Ala1125, Val1126, Leu1128, Asp1129, Tyr1130, Pro1131, Ala1132, Ser1135, Tyr1150, Gly1151, Asn1152, and Gly1153) were involved in hydrophobic interactions (Table 2).

Compound CD11575 had a GOLD fitness score of 68.67 and AutoDock binding energy –8.1 kcal/mol. However, X-Score binding energy of this compound was –6.8 kcal/mol (Table 1). Hydrogen bonds were formed by Asn1152, Gly1151, and Val1126 within the range of 2.39–3.09 Å, and hydrophobic interactions were formed by thirteen amino acids (His1051, Ile31123, Ala1125, Val1126, Leu1128, Asp1129, Tyr1130, Ser1135, Leu1149, Tyr1150, Gly1151, Asn1152, and Gly1153) of the active site with this compound within 2.42–3.90 Å (Table 2 and Figure 3).

Compound CD03173 presented to be a promising lead for the development of future drug candidates. Within the active site of the NS2B/NS3 enzyme system, three amino acids (Gly1133, Thr1134, and Ser1135) were involved in hydrogen bonds formation (2.56–3.26 Å) with this compound, and thirteen amino acids (Met1049, His1051, Ala1125, Val1126, Leu1128, Pro1131, Ala1132, Gly1133, Thr1134, Ser1135, Gly1151, Asn1152, and Gly1153) were involved in forming hydrophobic interactions within the range of 2.40–3.86 Å (Table 2 and Figure 3). This compound formed a stable complex with 72.01 GOLD fitness score and –8.7 kcal/mol binding energy (Table 1).

Compound JFD01698 had a –6.1 Kcal/mol AutoDock binding energy. However, GOLD fitness score and X-Score binding energy were found to be 71.21 and –6.3 kcal/mol, respectively (Table 1). Two amino acids (His1051 and Ser 1135) in the active site of the enzyme NS2B/NS3 formed hydrogen bonds within the range of 2.66–2.91 Å, and ten amino acids (His1051, Leu1128, Asp1129, Tyr1130, Pro1131, Ala1132, Gly1133, Thr1134, Ser1135, and Tyr1150) formed hydrophobic interactions within the range of 2.4.1–3.8.5 Å with this compound (Table 2 and Figure 3).

KM10383 had the lowest GOLD fitness score of 62.45 and lowest X-Score binding energy –5.9 kcal/mol (Table 1). AutoDock binding energy of this was –6.8 kcal/mol. Only two amino acids (His1051 and Ser1135) were involved in the formation of hydrogen bonds (2.55–3.09), and thirteen amino acids (His1051, Val1126, Leu1128, Asp1129, Tyr1130, Pro1131, Ala1132, Ser1135, Tyr1150, Gly1151, Asn1152, and Gly1153.) were involved in the formation of hydrophobic interactions with this compound (Table 2 and Figure 3). This compound formed the weakest association with the NS2B/NS3 protein, as compared to other complexes.

## 4. Conclusion

This study concludes seven compounds (CD11575, CD03173, HTS04601, HTS03171, HTS07252, JFD01698, and KM10383) as potential inhibitors against Zika virus protein NS2B/NS3 serine protease. Moreover, these compounds were also found to cover the drug-like properties. These inhibitors are commercially available and can be used for further experimental verification. The information obtained from this study can be used in the future for the development of more inhibitors against Zika virus.

## Figures and Tables

**Table 1:** Binding scores of the selected best compounds.

Compound	GOLD fitness	X-Score (kcal/mol)	AutoDock (kcal/mol)
Boronate	59.34	–5.5	–5.8
CD11575	68.67	–6.8	–8.1
HTS04601	69.59	–6.4	–7.4
HTS03171	67.81	–6.5	–6.9
HTS07252	69.68	–7.4	–7.5
JFD01698	71.21	–6.3	–6.1
CD03173	72.01	–7.2	–8.7
KM10383	62.45	–5.9	–6.8

**Table 2:** Crucial amino acids of the active site with their mode of interactions.

Compound	Hydrogen bonds	Range (Å)	Hydrophobic contacts	Range (Å)
Boronate	Lys1074	3.11	Lys1074, Thr1118, Ile1123, Ala1125, Val1126, Tyr1150, Gly1151, Asn1152, Gly1153	3.1–3.93
CD11575	Asn1152, Gly1153	2.33–2.82	His1051, Il31123, Ala1125, Val1126, Leu1128, Asp1129, Tyr1130, Pro1131, Ser1135, Leu1149, Tyr1150, Gly1151, Asn1152, Gly1153	2.42–3.90
CD03173	Gly1133, Thr1134, Ser1135,	2.56–3.26	Met 1049, His1051, Ala1125, Val1126, Leu1128, Tyr1130, Pro1131, Ala1132, Gly1133, Thr1134, Ser1135, Tyr1150, Gly1151, Asn1152, Gly1153	2.40–3.86
HTS03171	His1051, Gly1133, Ser1135, Gly1151	2.36–3.08	His1051, Ala1125, Val1126, Ala1127, Leu1128, Tyr1130, Pro1131, Ala1132, Gly1133, Thr1134, Ser1135, Tyr1150, Gly1151, Asn1152, Gly1153	2.34–3.89
HTS07252	His1051, Thr1134, Ser1135, Gly1151	2.60–3.14	Gln1035, Val1036, His1051, Ala1125, Val1126, Leu1128, Asp1129, Tyr1130, Pro1131, Ala1132, Gly1133, Thr1134, Ser1135, Ile1139, Tyr1150, Gly1151, Asn1152, Gly1153	2.27–3.87
JFD01698	His1051, Ser1135,	2.66–2.91	His1051, Leu1128, Asp1129, Tyr1130, Pro1131, Ala1132, Gly1133, Thr1134, Ser1135, Tyr1150	2.41–3.85
KM10383	His1051, Ser1135	2.55–3.09	His1051, Val1126, Leu1128, Asp1129, Tyr1130, Pro1131, Ala1132, Ser1135, Tyr1150, Gly1151, Asn1152, Gly1153	2.67–3.88
HTS04601	Val1126, Gly1151	2.72–2.88	His1051, Ala1125, Val1126, Leu1128, Asp1129, Tyr1130, Pro1131, Ala1132, Ser1135, Tyr1150, Gly1151, Asn1152, Gly1153	2.35–3.67

**Table 3:** Drug-like properties of the selected compounds.

Compound	MW	HBD	HBA	RtB	Log*P*
Boronate	508.4	7	9	13	
CD11575	470.034	1	5	8	5.0
HTS04601	425.507	3	7	7	3.75
HTS03171	511.041	1	7	8	4.03
HTS07252	475.548	3	9	7	1.69
JFD01698	403.191	2	5	4	2.31
CD03173	506.368	4	9	9	3.7
KM10383	443.587	1	6	9	2.21

MW: molecular weight; HBD: hydrogen bond donor; HBA: hydrogen bond acceptor; RtB: rotatable bond.

## Data Availability

The data used to support the findings of this study are included within the article.
